# Food related attention bias modification training for anorexia nervosa and its potential underpinning mechanisms

**DOI:** 10.1186/s40337-019-0276-9

**Published:** 2020-01-06

**Authors:** Daniela Mercado, Ulrike Schmidt, Owen G. O’Daly, Iain C. Campbell, Jessica Werthmann

**Affiliations:** 1 Section of Eating Disorders, Department of Psychological Medicine Institute of Psychiatry, Psychology and Neuroscience, King’s College London, London, UK; 2 Department of Neuroimaging, Institute of Psychiatry, Psychology & Neuroscience, King’s College London, London, UK; 3 Section of Eating Disorders, Department of Psychological Medicine Institute of Psychiatry, Psychology and Neuroscience, King’s College London, London, UK; 4 Department of Clinical Psychology and Psychotherapy, Institute of Psychology, Albert-Ludwigs University of Freiburg, Freiburg im Breisgau, Germany

**Keywords:** Eating disorders, Anorexia nervosa, Attention bias, Cognitive Bias, Attention Bias Modification.

## Abstract

Treatment outcomes in anorexia nervosa (AN) remain suboptimal, evidencing the need for better and more targeted treatments. Whilst the aetiology of AN is complex, cognitive processes such as attention bias (AB) have been proposed to contribute to maintaining food restriction behaviour. Attention bias modification raining (ABMT) has been investigated in other eating disorders (EDs) such as binge eating disorder (BED) as a means of modifying AB for food and of changing eating behaviour. Promising findings have been reported, but the mechanisms underlying ABMT are poorly understood. We hypothesise that in AN, ABMT has the potential to modify maladaptive eating behaviours related to anxiety around food and eating and propose two mechanistic models; (1) ABMT increases general attentional control (which will improve control over disorder-relevant thoughts) or (2) ABMT promotes stimulus re-evaluation. In this second case, the effects of ABMT might arise via changes in the subjective value of food stimuli (i.e. reward processing) or via habituation, with both resulting in a reduced threat response. Investigating the clinical potential of ABMT in AN holds the promise of a novel, evidence-based adjunctive treatment approach. Importantly, understanding ABMT’s underlying mechanisms will help tailor treatment protocols and improve understanding of the cognitive characteristics of AN and other EDs.

## Background

The involvement of cognitive mechanisms related to food processing, including food –related attention bias (AB) in the aetiology and maintenance of anorexia nervosa (AN) is well documented [1–3]. We propose that attention bias modification training (ABMT), a novel treatment approach, holds clinical potential for AN by modifying maladaptive AB for food. We also suggest that increasing knowledge of the neural/psychological processes involved in altering AB, will help maximize its clinical efficacy.

### Attention bias to food in AN

AB is a cognitive process whereby salient stimuli (e.g. food), selectively “capture” attention in comparison to neutral cues [4]. This can occur outside of conscious control and it is thought to influence subsequent behaviour, such as food consumption [5]. Cognitive models suggest that people with AN show aberrant attention processing of food cues (i.e. AB) because of their preoccupation with and/or fear of food. In support of this, a meta-analysis found AB to food pictures with a medium effect size in a mixed sample of eating disorder (ED) patients [1]. Eye-tracking data showed that while initial AB to food cues in people with AN was comparable to healthy controls, those with AN did not maintain attention on the food cues [2, 3]. Rather, they present attentional avoidance [2, 3]. This attentional avoidance pattern was more evident when they were presented with high caloric food cues, in contrast to low caloric food cues [3]. As research in healthy individuals indicates that duration of attentional gaze on food is related to subsequent craving and to food intake [6], it has been proposed that attentional avoidance of, rather than increased initial engagement with high caloric food cues, could be a strategy used to reinforce food restriction [7]. Attentional avoidance of high caloric food cues is mainly seen in adults with AN (longer illness duration) compared to adolescents (shorter illness duration) [3], suggesting that this behaviour becomes more ingrained as illness progresses and may be an important element in maintaining fear of food or restrictive eating behaviour.

### Attention bias modification training (ABMT)

ABMT is a form of cognitive bias modification training [8] with the potential for modifying AB triggered by different stimuli (e.g. food) [9]. Typically, ABMT is used to train attention towards disorder-incompatible stimuli in a relatively implicit way: it was developed by modifying the dot-probe paradigm used to assess AB [10]. In the original format of the task, participants are simultaneously presented with a disorder-relevant stimulus and a neutral stimulus on either side of a computer screen. Immediately after, a probe replaces one of the images and participants indicate the location of the probe as quickly as possible. AB is assumed when individuals respond faster when the probe replaces disorder- relevant stimuli. In the training version of the paradigm, the probe almost always (e.g. 95% of the time) replaces either the disorder-relevant or neutral stimulus, depending on the design. For example, if the aim of the training is to shift attention towards disorder-relevant cues, which would be the case for people with AN, the probe would be set to appear on the location of the threatening cue (e.g. high caloric food), manipulating attentional focus towards it, thereby reducing attentional avoidance.

While ABMT has been mainly used in the treatment of depression and anxiety disorders [11], a version involving training with food cues has been used in obesity and binge eating disorder (BED). Based on the hypothesis that sustained AB to food is associated with increased intake, the aim of ABMT in such studies was to reduce attention to food [7, 12]. In support of this, reviews and meta-analyses have described significant effects of different types of cognitive bias modification training (including ABMT) in changing AB and eating behaviour [13, 14].

### Theoretical models underpinning ABMT

Despite encouraging results on the potential therapeutic value of AMBT in problematic eating behaviour and different psychiatric disorders, findings are inconsistent. This may be because of limited understanding of the mechanisms underpinning ABMT’s potential therapeutic effect, which, in turn, may lead to heterogeneous and untargeted training designs that lack solid theoretical underpinnings. To maximise the potential of ABMT, it is necessary to establish the underlying mechanisms, and tailor the training according to differences between target populations. Some studies suggest ABMT’s effects are substantially due to increased general attentional control [11]. However, others suggest that its effects are mediated primarily by changes in stimulus evaluation [15]. These mechanisms are discussed below.

#### Attention control model

**Fig. 1:**
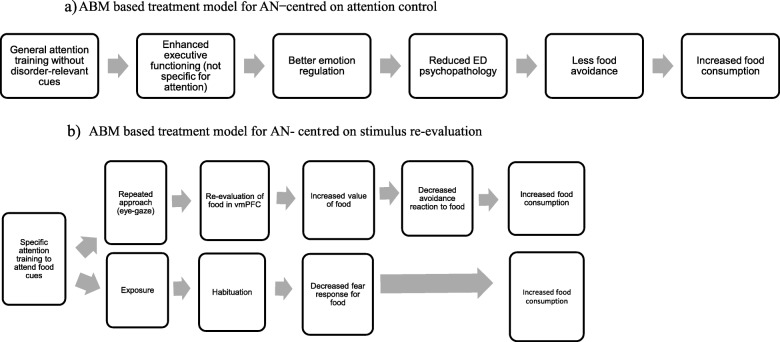
Theoretical models underpinning attention bias modification training (ABMT) for the treatment of anorexia nervosa (AN)

This proposes that an increase in general attention control improves control of disorder-relevant thoughts, i.e., strengthening higher order cortical processes will down-regulate emotion-relevant limbic structures. Thus, in AN, enhancing executive functioning will improve emotional control, and this is proposed to lead to a reduction in rumination and fear of food, which are part of ED psychopathology (Fig. 1a). In support of this, some studies in anxiety disorders indicate that training attention (regardless of contingency between cues and probes), improves attention control, and this improves participants’ control over anxiety-related cognitions and emotions [11]. However, others suggest that attention control might not be the only, or even the primary mechanism involved. Thus, Taylor et al. [16] showed that a reduction in both AB and anxiety symptoms only occurred in participants undertaking the “active” ABMT condition in comparison to a non-contingency training. Furthermore, a neuroimaging study reported decreased neural activation in areas implicated in threat-response after training people to look away from threat cues, compared to before ABMT [17], evidencing the involvement of stimulus-specific attention processes. These studies [16, 17] suggest that ABMT acts via modulation of circuits linked to attention control, specifically to executive and affective control (so called “top-down” processes), *and* via changes to stimulus-driven or “bottom-up” mechanisms to modify AB. The potential role of “bottom-up” mechanisms, would be consistent with a model which proposes that ABMT changes stimulus evaluation. This is discussed below.

#### Re-evaluation model

Here, ABMT is proposed to change the way the stimulus is perceived and/or evaluated. Two potential (not exclusive) mechanisms may be involved: (a) re-evaluation of stimuli, due to repeated approach or avoidance behaviour, changes the rewarding properties of the stimulus (repeatedly approaching high-calorie food, makes them more rewarding for people with AN), or, (b) habituation, associated with repeated exposure, changes the valence of the stimulus (repeatedly looking at high-calorie food, makes them less threatening).

In support of this, Goetz et al. [18] investigated whether biasing the attention of healthy controls (HCs) towards or away from rewarding words (e.g. rewarding foods) would implicitly prime the appetitive system. They reported that manipulating attention increased self-reported approach motivation and food intake. In a similar way, the subjective value of foods can be modified via cue-approach behaviour (go/no go training) without external reinforcement or any other explicit manipulation of value [15]: Pairing certain cues with a “go” response will increase the value of these items as a result of training a motor approach response toward them. Hence, these and other studies suggest that training attention towards or away from food stimuli changes eating behaviour by altering the subjective value of food. If so, patients with AN would re-evaluate food cues more positively by repeatedly having to approach food with their eye-gaze. This change in approach behaviour should counter the attentional avoidance of food cues in people with AN, and in time, might change food intake (Fig. 1b).

An alternative explanation for a re-evaluative effect associated with ABMT may be related to repeated exposure to disorder-relevant cues. Exposure based on habituation models assumes a disruption of the fear avoidance reaction by learning that the feared consequences will not occur, leading to fear reduction and formation of new associations with the stimuli [19]. The premise is that ABMT is effective due to habituation and to a reduced fear response towards relevant stimuli (i.e. food), hence, food cues become less likely to trigger an avoidance response. This warrants investigation, since no studies have explored the role of exposure as a contributory mechanism in ABMT. In addition, if exposure proves to be an underpinning mechanism of ABMT, it would be worth exploring different approaches to the training e.g. using inhibition-learning instead of habituation to determine the best way to achieve a longer-term effect of exposure [19].

## Conclusion

Testing the proposed mechanistic models of ABMT in AN and investigating their clinical efficacy has the potential of providing a novel treatment approach for AN. It will also contribute to our understanding of cognitive patterns that underlie some of the maladaptive behaviours in AN, e.g. food restriction and fear of food. Lastly, a better understanding of mechanisms and active components of add-on interventions such as ABMT, will help improve treatments for AN.

## Data Availability

n/a

## Abbreviations

AB: Attention bias
ABMT: Attention Bias Modification Training
AN: Anorexia Nervosa
BED: Binge Eating Disorder
ED: Eating disorder
HC: Healthy controls
